# The Effect of Cold Temperature on Increased Exacerbation of Chronic Obstructive Pulmonary Disease: A Nationwide Study

**DOI:** 10.1371/journal.pone.0057066

**Published:** 2013-03-15

**Authors:** Ching-Min Tseng, Yung-Tai Chen, Shuo-Ming Ou, Yi-Han Hsiao, Szu-Yuan Li, Shuu-Jiun Wang, Albert C. Yang, Tzeng-Ji Chen, Diahn-Warng Perng

**Affiliations:** 1 Department of Chest Medicine, Taipei Veterans General Hospital, Taipei, Taiwan; 2 Department of Medicine, Taipei City Hospital Heping Fuyou Branch, Taipei, Taiwan; 3 Division of Nephrology, Department of Medicine, Taipei Veterans General Hospital, Taipei, Taiwan; 4 Institute of Clinical of Medicine, National Yang-Ming University, Taipei, Taiwan; 5 Department of Neurology, Neurological Institute, Taipei Veterans General Hospital, Taipei, Taiwan; 6 Department of Psychiatry, Taipei Veterans General Hospital, Taipei, Taiwan; 7 Department of Family Medicine, Taipei Veterans General Hospital, Taipei, Taiwan; 8 School of Medicine, National Yang-Ming University, Taipei, Taiwan; Wadsworth Center, United States of Amerida

## Abstract

**Background:**

Seasonal variations in the acute exacerbation of chronic obstructive pulmonary disease (COPD) have been reported. However, the influence of air temperature and other meteorological factors on COPD exacerbation remains unclear.

**Methods:**

National Health Insurance registry data from January 1, 1999 to December 1, 2009 and meteorological variables from the Taiwan Central Weather Bureau for the same period were analyzed. A case-crossover study design was used to investigate the association between COPD exacerbation and meteorological variables.

**Results:**

A total of 16,254 cases who suffered from COPD exacerbation were enrolled. We found that a 1°C decrease in air temperature was associated with a 0.8% increase in the exacerbation rate on event-days (95% confidence interval (CI), 1.015–1.138, *p* = 0.015). With a 5°C decrease in mean temperature, the cold temperature (28-day average temperature) had a long-term effect on the exacerbation of COPD (odds ratio (OR), 1.106, 95% CI 1.063–1.152, *p*<0.001). In addition, elderly patients and those who did not receive inhaled medication tended to suffer an exacerbation when the mean temperature dropped 5°C. Higher barometric pressure, more hours of sunshine, and lower humidity were associated with an increase in COPD exacerbation.

**Conclusions:**

This study demonstrated the effect of cold temperatures on the COPD exacerbation rate. Elderly patients and those without inhaled medicine before the exacerbation event were affected significantly by lower mean temperatures. A more comprehensive program to prevent cold stress in COPD patients may lead to a reduction in the exacerbations rate of COPD.

## Introduction

Exacerbation of chronic obstructive pulmonary disease (COPD), defined as an acute worsening of respiratory symptoms beyond the usual status [1], leads to a deterioration in lung function [2] and quality of life [3] and an increased risk of mortality [4]. Reducing the burden of COPD requires better prevention, timely recognition, and effective management of exacerbations. Early identification of risk factors for exacerbation may help to prevent high-risk COPD patients from suffering an exacerbation and improve their quality of life. Exacerbation frequency in COPD is associated with several precipitating factors, including older age, lower body mass index, poor lung function, history of prior exacerbations and co-morbidities [5]. Exposure to some environmental factors such as air pollutants, occupational hazards and infections may cause exacerbation [6], but air temperature as a risk factor is not well understood.

COPD patients have been reported to experience more frequent exacerbation and have higher hospitalization and morbidity rates during the winter season [5], [7]. In addition, respiratory viral infections have seasonal variations and are more prevalent in the winter [8]–[10]. Meteorological factors, such as temperature, humidity, wind speed, sunshine and rainfall tend to vary with different seasons. Although COPD exacerbations are more frequent in the winter, it is unclear whether the season itself or the above-mentioned meteorological factors exert an influence on exacerbations.

Taiwan, located in the western Pacific Ocean, is a small mountainous island, 394 km long and 144 km wide at its broadest point, on the Tropic of Cancer [11]. The comprehensive coverage offered by the Taiwanese national health insurance program provides an opportunity to investigate the linkage between meteorological effects, seasonal changes and exacerbations of COPD.

The aims of this study were to investigate meteorological variables in relation to exacerbation rates of COPD, and the potential protective effects of vaccination or inhaled medicine on COPD patients. The results could provide detailed information to assist in the prevention of exacerbation of COPD during the winter season.

## Materials and Methods

### Data source

A National Health Insurance (NHI) program was launched in 1995 in Taiwan. To date, enrollment in this program is mandatory and coverage is approximately 98%. In 1999, the Bureau of National Health Insurance began to release patient data in electronic form under the National Health Insurance Research Database (NHIRD) project. Various extracted datasets are available to researchers, and hundreds of published papers have used these data for their studies. Data from the NHIRD was analyzed for the current study. The hospital's ethics committee approved the study. The diseases were coded in the NHIRD using the coding of the ICD-9-CM, 2001 edition. In this study, the National Health Research Institute (NHRI) randomly sampled a representative database of 1,000,000 patients from the year 2000 registry of all NHI enrollees using a systematic sampling method for research purposes, known as the Longitudinal Health Insurance Database (LHID). There were no statistically significant differences in age, sex, or health care costs between the sample group and all enrollees, as reported by the NHRI. We used the admissions and outpatient visits databases of the sample cohort, both of which included information on patient characteristics, including sex, date of birth, date of admission, date of discharge, date of visit, and up to 5 discharge diagnoses or 3 outpatient visit diagnoses. The data files also contained information on patient prescriptions, including the names of prescribed drugs, dosage, duration, and total expenditure.

### Meteorological data

In Taiwan, summer is defined as the warmest months of June, July and August, and winter is the coldest months of December, January, and February. The 2 seasons of autumn (September to November) and spring (March to May) are the transition periods between the warmest and coldest months [12]. We collected primary weather variables, including average daily ambient temperature, daily maximum ambient temperature, daily minimum ambient temperature, average daily relative humidity, average daily barometric pressure, maximum wind speed, and total hours of sunshine on each day, from all 14 weather stations in Taiwan. Local meteorological weather data measured at corresponding times in the local weather stations were provided by the Taiwan Central Weather Bureau. A gradient change for each weather time series was also derived, defined as the difference in the average of a weather variable from the previous day.

### Study design and patient selection

We conducted a case-crossover study design to examine the effect of short and transient exposures on acute outcomes [13]. Each patient served as his or her own control; i.e., the study subjects were self-matched. The distribution of exposure (meteorological conditions) was compared between cases and controls. The difference between our case-crossover study and a case-control study is that the exposure at the time just prior to the event (the case period) was compared with a set of referent periods (control periods), or the expected distribution of exposure for non-event follow-up times. The case period in our study was defined as the date of admission. For the referent period selection, we used the ambidirectional approach, which was proved consistent and as powerful as the time-stratified approach [14]. Referent periods were chosen as the exposure one month before the admission date and one month after the admission date for each case. Symmetric referent periods were selected to account for potential biases from linear time trends of temperature exposure [15].

From January 1, 1999 to December 1, 2009, all patients older than 40 years who had a diagnosis of exacerbation of COPD (ICD-9 CM code 491.21) were identified. According to the definition of exacerbation of COPD [16], we further validated the diagnosis of COPD exacerbation by patients receiving treatments with antibiotics, bronchodilator or intravascular/oral steroids. The index date was the date of outpatient visit, emergency room visit, or admission. The control date was selected as one month before the index date and one month after the index date. Long-term inhaled medicine use and vaccinations were recorded. Inhaled medicine was defined as a prescription for an inhaled long-acting ß_2_ agonist (LABA), long-acting muscarinic antagonist (LAMA) or/and inhaled corticoid steroid (ICS) for more than 28 days within 6 months before the index date. Vaccination included influenza and pneumococcus vaccines received within one year before the index date.

### Statistical analysis

Normally distributed continuous data were expressed as means ± standard deviations (SD). Numeric data with non-normal distributions were expressed as medians and inter-quartile ranges. Pearson χ^2^ tests were carried out for categorical variables, and the independent t-test and Mann-Whitney U test were used for parametric and nonparametric continuous variables, respectively. For the case- crossover analyses, the conditional logistic regression model was used to estimate the odds ratios (ORs) and the 95% confidence intervals (CIs). We performed subgroup analyses by stratifying the various characteristics of the patients, including age, sex, vaccination, and use of inhaled medicine. All analyses were conducted using STATA statistical software (version 11.0, STATA Corp).

## Results

### Demographics and meteorological variables

A total of 18,454 cases of COPD exacerbation were identified in our study cohort during an 11-year period. After excluding patients who were under 40 years (n = 262) and those who had a repeat of the same episode within one month after discharge (n = 1938), we enrolled 16,254 cases for analysis. Study subjects were predominantly male (77.4%) and the mean age was 75.5±10.2 years. Hypertension (80.2%), coronary artery disease (61.0%) and cerebral vascular disease (49.3%) were the most common co-morbidities (Table 1). In terms of meteorological variables, the annual temperature was 23.8±4.7°C, with an average daily temperature range of 7.3–33.3°C and mean temperature variation of 7.1±2.5°C. Relative humidity ranged from 34–100% and barometric pressure was 1008.7±7.1 hPa (Table 2). Relative humidity was lower in winter in Taiwan (mean humidity in summer: 78.01%, SD 7.1%, range: 50–100%; in winter: 75.36%, SD 8.6%, range: 35–100%).

**Table 1:** Demographic data of exacerbation of chronic obstructive pulmonary disease cases from 1999 to 2009 in Taiwan.

Case number	N = 16,254
**Age, mean ± SD**	75.5±10.2
**Male, %**	77.4% (12,577)
**Diabetes, %**	42.4% (6,898)
**Hypertension, %**	80.2% (13,032)
**Heart Failure, %**	26.0% (4,224)
**Cerebral vascular disease, %**	49.3% (8,015)
**Coronary artery disease, %**	61.0% (9,922)
**Chronic kidney disease, %**	28.7% (4,665)
**Received vaccination within 1 year, %**	29.5% (4,794)
**Received inhaled medicine** * **, %**	8.7% (1,408)

* Inhaled medicine included: inhaled long-acting ß_2_ agonist, long-acting muscarinic antagonist or/and inhaled corticosteroids.

**Table 2:** Meteorological variables from 1999 to 2009 in Taiwan.

Variables		Range
**Temperature, mean±SD**	23.8±4.7	7.3–33.3
**Temperature, median (IQR)**	24.8 (20.2–27.8)	7.3–33.3
**Temperature variation, mean±SD**	7.1±2.5	0.6–19.8
**Temperature variation, median (IQR)**	7.1 (5.4–8.7)	0.6–19.8
**Relative humidity %, mean±SD**	76.1±7.8	34–100
**Relative humidity %, median (IQR)**	76 (71–81)	34–100
**Wind speed m/s, mean±SD**	2.4±1.3	0–15.0
**Wind speed m/s, median (IQR)**	2.0 (0–0.9)	0–15.0
**Barometric pressure, hPa, mean±SD**	1008.7±7.1	962.7–1031.7
**Barometric pressure, hPa, median (IQR)**	1008.7 (1004.1–1013.7)	962.7–1031.7
**Sunshine, HR/day, mean±SD**	5.3±4.9	0–13.2
**Rainfall days in consecutive 7-days**	2.0 (0–4)	
**Rainfall days in consecutive 14-days**	4.0 (0–7)	

SD: Standard deviation; IQR: Interquartile range.

### Association of meteorological variables and COPD exacerbation

**Figure 1:**
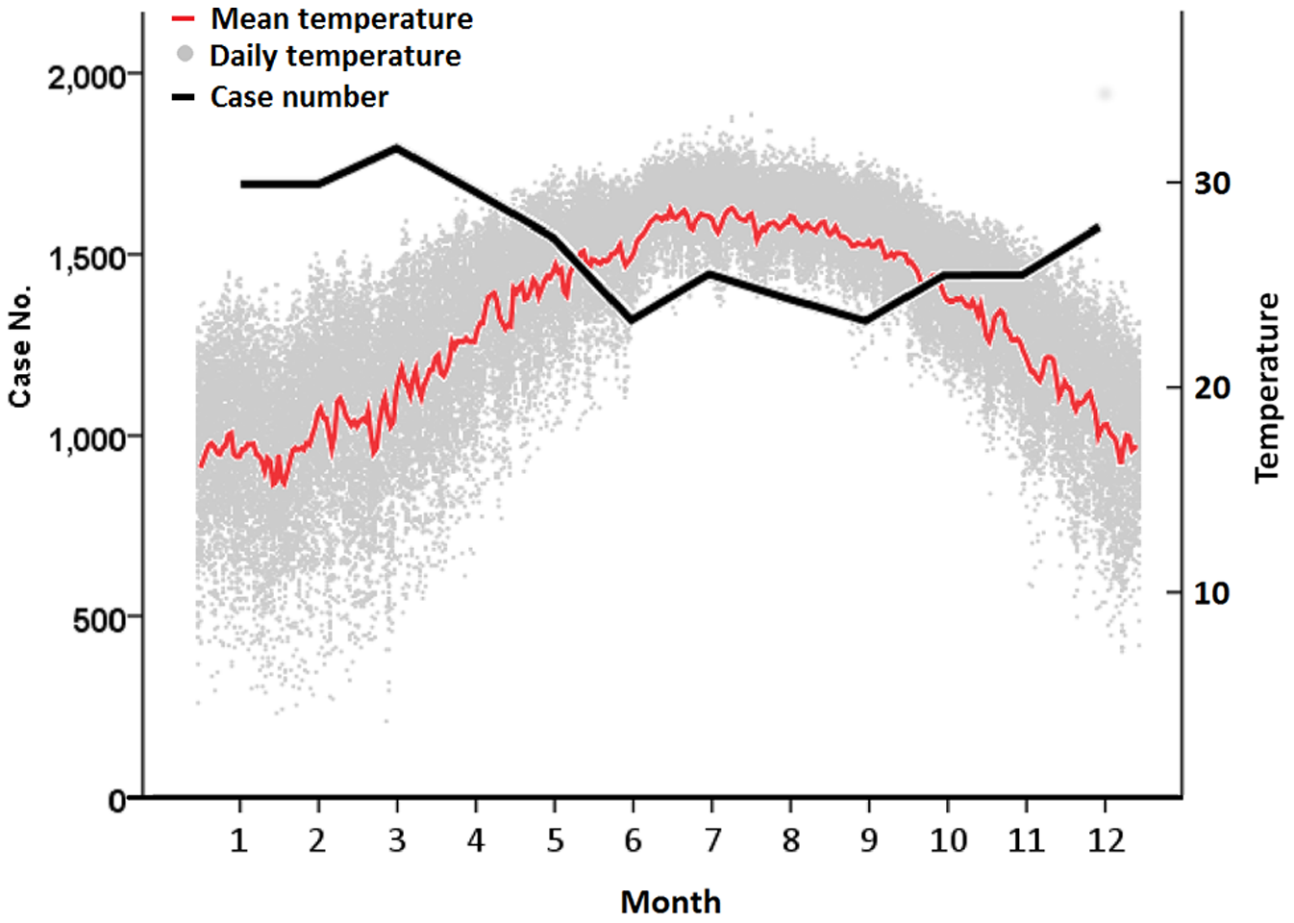
Monthly cases of exacerbation of chronic obstructive pulmonary disease and air temperature in Taiwan.

The accumulated monthly cases of COPD exacerbation in association with air temperature are presented in Figure 1. The mean temperature in the winter months (December to February) and summer months (June to August) was 17.9±3.3°C and 28.4±1.5°C, respectively. The observed numbers of monthly cases were higher in the winter than in the summer.

**Figure 2:**
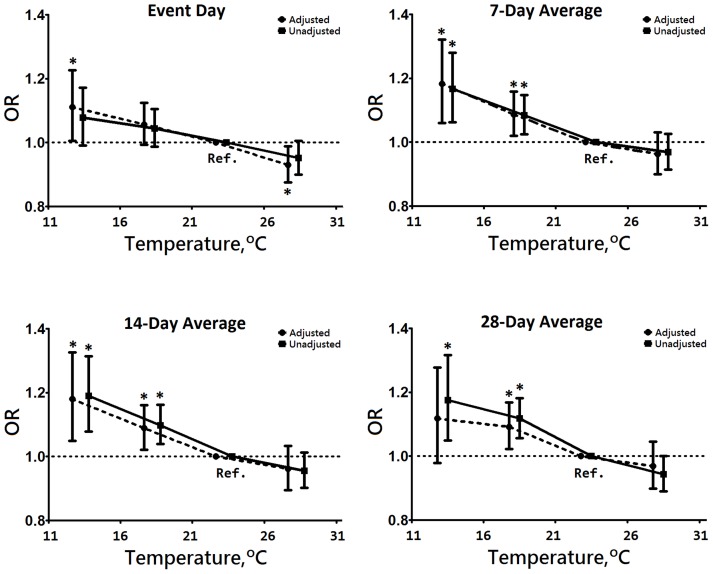
Odds ratios of exacerbation of chronic obstructive pulmonary disease relative to the entire range of mean temperatures. * ***P*** **<0.05.**

Statistical analysis of the case-control crossover model in terms of meteorological data on event-day, and 3-day, 7-day, 14-day and 28-day averages were carried out (Table 3). Estimated ORs and corresponding 95% CIs for the effect of exacerbation events are expressed in terms of a 5°C decrease in mean temperature, because this represents a plausible change in temperature; the SD of the mean temperature was 4.7°C in Taiwan. We found that a 1°C decrease in mean temperature was significantly associated with a 0.8% increase in cases of COPD exacerbation (95% CI 1.015–1.138, *p* = 0.015) on event-days. A decrease of 5°C in mean temperature was significantly correlated with increased numbers of exacerbations from event-days (OR, 1.039, 95% CI 1.007–1.071, *p* = 0.015) to 28 days (OR, 1.106, 95% CI 1.063–1.152, *p*<0.001). Nevertheless, temperature variation had a shorter correlation, within 7 days only (OR, 1.109–1.016). Other meteorological factors, such as barometric pressure, had a positive correlation with the increased exacerbation rate from event-days (OR, 1.005, 95% CI 1.000–1.009, *p* = 0.036) to 28 days (OR, 1.014, 95% CI 1.007–1.020, *p*<0.001). Relative humidity was negatively, and sunshine hours were positively correlated with increased numbers of COPD exacerbations on event-days and within 7 days (Table 3). When evaluating mean temperature after adjusting relative humidity, barometric pressure, wind speed, duration of sunshine and rainfall days, a stratified-temperature in relation to exacerbation rate from event-days to 28-days is shown (Figure 2).

**Table 3:** Odds ratios of exacerbation of chronic obstructive pulmonary disease in relation to meteorological variables.

	Event day			3-day Average*			7-day Average**			14-day Average+			28-day Average++		
	OR	95% CI	*p* Value	OR	95% CI	*p* Value	OR	95% CI	*p* Value	OR	95% CI	*p* Value	OR	95% CI	*p* Value
**Mean temperature** #	1.039	1.007–1.071	0.015	1.055	1.021–1.089	0.001	1.075	1.038–1.114	<0.001	1.099	1.058–1.141	<0.001	1.106	1.063–1.152	<0.001
**Temperature variation**	1.009	1.001–1.017	0.040	1.014	1.003–1.025	0.016	1.016	1.002–1.031	0.029	1.011	0.993–1.030	0.218	1.011	0.987–1.035	0.365
**Relative humidity,%**	0.996	0.994–0.999	0.003	0.996	0.993–0.999	0.004	0.995	0.991–0.998	0.005	0.998	0.993–1.002	0.346	1.001	0.996–1.007	0.662
**Barometric pressure**	1.005	1.000–1.009	0.036	1.005	1.001–1.010	0.030	1.008	1.003–1.014	0.003	1.011	1.005–1.017	<0.001	1.014	1.007–1.020	<0.001
**Wind speed, m/s**	1.007	0.989–1.026	0.429	1.007	0.983–1.031	0.574	1.021	0.989–1.054	0.194	1.025	0.985–1.067	0.221	1.029	0.979–1.082	0.255
**Sunshine, hours/day**	1.007	1.001–1.012	0.012	1.008	1.001–1.015	0.022	1.009	1.000–1.018	0.041	1.003	0.992–1.015	0.593	0.987	0.972–1.003	0.110

* Mean meteorological data of the same day and 2 previous days.

** Mean meteorological data of the same day and 6 previous days.

+ Mean meteorological data of the same day and 13 previous days.

++ Mean meteorological data of the same day and 27 previous days.

# Decrease per 5°C.

### Temperature effect on exacerbation rate in relation to stratified subgroup factors

To evaluate the association between temperature and exacerbation in different subpopulations, we performed subgroup analyses stratified by age, sex, vaccination and inhaled medicine treatment. The association between a mean temperature decrease of 5°C and an increased rate of COPD exacerbation was more significant in the elderly and in those without inhaled medication within one month (Table 4). There was no difference between patients with or without vaccination.

**Table 4:** Odds ratios of exacerbation of chronic obstructive pulmonary disease with a 5°C decrease in mean temperature# stratified by age, sex, vaccination and inhaled medicine.

	Event day			3-day Average			7-day Average			14-day Average			28-day Average		
	OR	95% CI	*p* Value	OR	95% CI	*p* Value	OR	95% CI	*p* Value	OR	95% CI	*p* Value	OR	95% CI	*p* Value
**Age≧65**	1.073	1.022–1.126	0.005	1.098	1.042–1.158	0.001	1.137	1.069–1.210	<0.001	1.153	1.073–1.239	<0.001	1.134	1.040–1.237	0.005
**Age<65**	1.029	0.919–1.154	0.618	1.079	0.954–1.220	0.225	1.043	0.903–1.204	0.568	1.109	0.940–1.308	0.220	1.134	1.040–1.237	0.005
**Male**	1.056	1.004–1.111	0.036	1.084	1.025–1.145	0.004	1.113	1.043–1.188	0.001	1.141	1.058–1.230	0.001	1.120	1.022–1.226	0.015
**Female**	1.110	1.009–1.221	0.033	1.143	1.030–1.268	0.012	1.161	1.028–1.310	0.016	1.169	1.017–1.344	0.028	1.075	0.910–1.271	0.395
**Received vaccination** +	1.054	0.972–1.143	0.200	1.075	0.984–1.173	0.108	1.158	1.043–1.286	0.006	1.221	1.081–1.380	0.001	1.181	1.018–1.371	0.028
**Without received vaccination** +	1.074	1.018–1.134	0.010	1.107	1.044–1.174	0.001	1.112	1.039–1.191	0.002	1.119	1.034–1.211	0.005	1.084	0.986–1.192	0.095
**Received inhaled medicine** *	0.998	0.819–1.216	0.981	1.036	0.932–1.290	0.751	1.044	0.806–1.353	0.742	1.126	0.826–1.535	0.453	1.091	0.740–1.608	0.659
**Without inhaled medicine** *	1.070	1.020–1.122	0.006	1.105	1.049–1.164	<0.001	1.127	1.060–1.198	<0.001	1.146	1.068–1.229	<0.001	1.110	1.020–1.208	0.016

# Adjusted for relative humidity, barometric pressure, wind speed, and duration of sunshine.

* Received inhaled medicine, including inhaled long-acting ß_2_ agonist, long-acting muscarinic antagonist or/and inhaled corticosteroid for more than 28 days within 6 months before the index day.

+ Received vaccination within one year before the event.

## Discussion

Our study demonstrated the effect of mean temperature on the exacerbation of COPD. A 1°C decrease in air temperature was associated with a 0.8% increase in the exacerbation rate. The Long-term (28-day average) cold temperatures increased the risk of the exacerbation of COPD. In addition, elderly patients and those who did not receive inhaled medication tended to experience exacerbation when the mean temperature dropped 5°C. Influenza and pneumococcal vaccines did not influence the decreased exacerbation rate when there was a decrease of 5°C in mean temperature. Higher barometric pressure, more hours of sunshine and lower humidity were associated with an increase in COPD exacerbation. Temperature appears to be a potential risk factor for exacerbation.

It has been reported that there was a 2-fold increase in the COPD exacerbation rate in winter [5], [7]. Neither of these studies identified the effect of temperature on the exacerbation. Ferrari et al found that the increase in daily consultations for COPD patients was associated with a decrease of 0.72 k in temperature [17]. The lag effect of cold temperature on mortality has been reported. A 1°C decrease in temperature was associated with a 1.35% (95% CI: 1.16–1.53) increase in the daily total number of natural deaths and a 3.30% (95% CI: 2.61–3.99) increase in respiratory deaths [18]. This cold effect on mortality persisted up to 23 days [18]. These results are compatible with our findings that a lower mean temperature predicted a higher exacerbation rate and that the effects of cold temperature on the exacerbation of COPD may be longer-running. The possible explanations for the exacerbation attributed to cold temperatures included increased exposure to viral infection [19], reduced daily physical activities [7], and a direct cold temperature effect on broncho-constriction [20]. Donaldson et al reported that temperature was related to a reduction in lung function and that it contributed to increased COPD exacerbation [21]. Furthermore, there were some possible explanations for the long-term cold temperature effect. Exposure to cold temperatures could rapidly reduce the immunity to respiratory infections [22] and decrease mucociliary clearance [23], the consequence of which is that a progression to a full-blown exacerbation of COPD may take a long time.

An early study reported that the combination of a higher temperature and a rise in barometric pressure predicted deterioration in the respiratory status of patients with chronic lung disease [24]. In Bavaria, Germany, barometric pressure has been shown to be positively correlated with an increase in daily COPD consultations, but solar radiation and humidity showed negative correlations [17]. Our study revealed that barometric pressure had a positive correlation with the exacerbation rate, but that humidity and sunshine hours were negatively associated with this same rate. Increased humidity may eliminate the risk of a triggering of COPD exacerbation. Rea et al reported that long-term humidification therapy significantly reduced exacerbation days, increased the time to first exacerbation and improved lung function and quality of life in patients with COPD and bronchiectasis. Humidity might be a natural way to prevent exacerbation [25]. The results related to sunshine factor in our study showed reverse results compared with the previous study [17]. Different measurements and latitudes or geographic statuses may contribute to the differences in results. Further research on sunshine exposure and COPD exacerbation may be needed.

COPD patients who received LABA, LAMA or a combination with ICS were less affected by cold temperatures. A number of studies showed that inhaled medicine could reduce the frequency of exacerbation [26]–[28]. This is the first study to show that these inhaled medications could reduce the impact of cold temperature-related exacerbation of COPD. With regard to the age factor, elderly patients had a higher risk of exacerbation when the mean temperature dropped 5°C. Lindemann et al reported that nasal complaints in elderly patients were a consequence of lower intranasal air temperature and humidity values [29]. The progressive degradation of physiological functions in the elderly increases the risk of the harmful effects of cold exposure [30]. Elderly patients should be careful about preventing cold stress.

Although this study showed that the rate of COPD exacerbation when the mean temperature decreased 5°C was not reduced among patients who received vaccination, the prevention of lower respiratory tract infection by the use of influenza and pneumococcal vaccine is still recommended in the global guidelines [31], [32]. It has been reported that respiratory viruses were more prevalent in the winter [7], including human rhinoviruses, respiratory syncytial viruses and others [10]. Tillett et al reported that a close relationship existed between excess winter-season mortality and influenza-like illness that was independent of the cold temperature effect [33]. Influenza vaccine did reduce serious illness and mortality [34]. In this study, the 29.5% of patients with COPD exacerbation who received vaccination may not reflect the whole picture of the preventive effect of influenza vaccine.

The limitations of this study included the lack of some relevant information in the database, such as exposure to air pollutants, indoor temperature, socioeconomic status, or the temperature exposure of the individuals. Viral epidemiology was also not available in our dataset. The temperature data provided by weather stations in the countryside may not reflect the effect of temperatures on COPD exacerbation in urban areas.

In conclusion, this study demonstrated the effect of cold temperatures on the COPD exacerbation rate. Elderly patients and those without inhaled medicine before the exacerbation event were affected significantly by lower mean temperatures. A more comprehensive prevention of cold stress with regard to COPD patients may lead to a reduction in the exacerbation rate of COPD.
